# Examining the distributional equity of urban tree canopy cover and ecosystem services across United States cities

**DOI:** 10.1371/journal.pone.0228499

**Published:** 2020-02-11

**Authors:** Christopher B. Riley, Mary M. Gardiner

**Affiliations:** Department of Entomology, The Ohio State University, Columbus, Ohio, United States of America; National Institute of Geophysics, Geodesy and Geography, BULGARIA

## Abstract

Examining the distributional equity of urban tree canopy cover (UTCC) has increasingly become an important interdisciplinary focus of ecologists and social scientists working within the field of environmental justice. However, while UTCC may serve as a useful proxy for the benefits provided by the urban forest, it is ultimately not a direct measure. In this study, we quantified the monetary value of multiple ecosystem services (ESD) provisioned by urban forests across nine U.S. cities. Next, we examined the distributional equity of UTCC and ESD using a number of commonly investigated socioeconomic variables. Based on trends in the literature, we predicted that UTCC and ESD would be positively associated with the variables median income and percent with an undergraduate degree and negatively associated with the variables percent minority, percent poverty, percent without a high school degree, percent renters, median year home built, and population density. We also predicted that there would be differences in the relationships between each response variable (UTCC and ESD) and the suite of socioeconomic predictor variables examined because of differences in how each response variable is derived. We utilized methods promoted within the environmental justice literature, including a multi-city comparative analysis, the incorporation of high-resolution social and environmental datasets, and the use of spatially explicit models. Patterns between the socioeconomic variables and UTCC and ESD did not consistently support our predictions, highlighting that inequities are generally not universal but rather context dependent. Our results also illustrated that although the variables UTCC and ESD had largely similar relationships with the predictor variables, differences did occur between them. Future distributional equity research should move beyond the use of proxies for environmental amenities when possible while making sure to consider that the use of ecosystem service estimates may result in different patterns with socioeconomic variables of interest. Based on our findings, we conclude that understanding and remedying the challenges associated with inequities requires an understanding of the local social-ecological system if larger sustainability goals are to be achieved.

## Introduction

Urban forests are an integral green infrastructure component that carry out many critical ecological functions, including sequestering carbon and producing oxygen during photosynthesis and supporting wildlife with the resulting biomass [1–4]. When humans benefit or are harmed by these functions, they are often referred to as ecosystem services or disservices, respectively [5–8]. Urban forests provide regulating ecosystem services such as carbon sequestration and storage, atmospheric pollution removal, mitigation of the urban heat island effect, and stormwater runoff reduction, in addition to other social, cultural, and human-health related benefits [7,9–13]. Ecosystem disservices such as damage to infrastructure or the deposition of allergenic pollen can also result [6,8,14]. Despite these potential liabilities, urban forests are widely considered an environmental amenity due to the many benefits they confer [15]. Examining the distributional equity of urban tree canopy cover (UTCC) has increasingly become an important interdisciplinary focus of ecologists and social scientists (Table 1) [15–22]. However, while UTCC may serve as a useful proxy, it is ultimately not a direct measure of the ecosystem services provided by trees [23–26].

**Table 1:** Studies examining associations between indicators of urban vegetation presence or abundance and socioeconomic variables. Our predictions are based on the prevailing patterns supported in the literature.

Socioeconomic Variables	Predicted Association with UTCC	Evidence Supporting Prediction	Evidence in Opposition to Prediction	No Pattern
Median Income	Positive	[15,17,19–21,27–44]	[21,45]	[22]
Percent Poverty	Negative	[28,31,41,46–48]		
Percent Minority	Negative	[15,16,18,19,27–30,35,37,41,45,46,48]	[15,20,21,28,38,45,46]	
Percent without a High School Degree	Negative	[19,21,27,28,38,45]		[20]
Percent with an Undergraduate Degree	Positive	[21,22,43–45]	[45]	
Median Year Home Built	Negative	[22,27,29,38,40,49,50]	[21,34]	[31,43]
Percent Renters	Negative	[16,18,21,28,37,40,42,43,46]	[45]	[29]
Population Density	Negative	[17,18,21,27,30,38–40,51]		[22]

Advances in ecosystem modeling have allowed for the derivation of quantitative estimates of the benefits provided by urban forests occurring in distinct geographic regions based on UTCC estimates and other local data [1,9,52]. Ground-based inventories that generate forest structural data form the foundation for many calculations of forest function [1]. These in tandem with data describing the abiotic environment, broader ecological community, and biology of the species of interest can all inform models of an urban forest’s biogeochemical pools and fluxes [52]. However, when the emphasis shifts from calculating ecosystem functions to services, and in particular their monetary values, it becomes necessary to incorporate additional parameters associated with the local human population (e.g. population density, economic conditions). Based on these distinctions, two cities could conceivably have structurally similar forests that carry out a function such as carbon sequestration at comparable rates, yet divergent estimates of the monetary value associated with the service due to different human contexts. Thus, equivalent levels of UTCC have the potential to provide differing levels of ecosystem services from one geographic area to the next.

A number of tools have been developed in recent years that attempt to quantify ecosystem services and their value [53]. One of the most widely used for urban forests is the i-Tree platform (https://www.itreetools.org/). i-Tree is a publicly available suite of software programs and applications backed by peer-reviewed research that allows users to obtain estimates of the ecosystem services and monetary value of urban forests based on a variety of data collection techniques. For example, i-Tree Eco (previously the Urban Forest Effects (UFORE) model [1]), which requires ground survey data, has been used to compare urban forest communities and their benefits across land use types and to estimate the expected value added through large-scale tree planting initiatives [9,54]. Using ground survey data, i-Tree STRATUM (currently named i-Tree Streets) was used to estimate the ecosystem services and value of street trees in Lisbon, Portugal while i-Tree Canopy was used to estimate UTCC for Williamstown (a suburb of Melbourne), Australia entirely through random point sampling on Google Maps [55,56]. When combined with data related to the local human population, these tools have the potential to highlight inequities in the distribution of ecosystem services based on socioeconomic status. For example, the UFORE model was used to document the inequitable provision of the ecosystem services of air pollution removal and energy savings through shading among White, African American, and Hispanic areas within Miami-Dade County, Florida [57].

Recent paradigm shifts within the field of urban ecology call for interdisciplinary research approaches that view cities as social-ecological systems [58,59]. Ecology *for* the city (instead of *in* or *of*) highlights an important new focus of the field described as “shaping city form and function to create better outcomes for people and places” [58]. Integrative environmental justice studies examining the distribution of UTCC in relation to socioeconomic patterns often fall within the ecology *for* the city paradigm. These studies seek to understand both the social and ecological dimensions of the urban ecosystem and advance sustainability goals through increased and equitably distributed UTCC. To date, a substantive body of research has documented inequities in the distribution of UTCC, and urban vegetation abundance more generally (Table 1). Some of the most consistent patterns to emerge have been positive associations between advantaged populations (e.g. high income, college educated) and negative associations between disadvantaged or marginalized populations (e.g. low income, racial and ethnic minorities, no high school degree) and these environmental amenities (Table 1).

Efforts to understand if patterns of environmental inequity are universal across urban areas have led to important advancements in methodologies. Shifts from case studies to comparative, multi-location or large scale investigations [60–62], the incorporation of high-resolution datasets [49,50], and the use of more sophisticated analytical methodologies [15,18,21,33,37,63] are all approaches that aim to improve our understanding of underlying patterns of environmental injustice. Schwarz et al. (2015) employed these strategies to examine the distributional equity of UTCC in relation to income and race/ethnicity across seven U.S. cities. The researchers utilized high-resolution UTCC and socioeconomic data and moved beyond conventional correlation or regression analyses by using spatial autoregressive models [15]. In doing so, Schwarz et al. (2015) determined that the more robust spatial models often decreased or eliminated the significance of the socioeconomic predictors of UTCC. While acknowledging some caveats and limitations of UTCC distributional equity studies (Table 2), their research demonstrates the value of utilizing high-resolution datasets in conjunction with spatially explicit models in a multi-city framework to understand the pervasiveness of environmental inequities and identify where efforts are needed to address them [15].

**Table 2:** Caveats to consider when carrying out or interpreting results from urban tree canopy cover distributional equity studies. Reproduced with modifications from Schwarz et al. (2015), with permission from K. Schwarz.

**1**	Variables not examined might be better predictors of urban tree canopy cover or ecosystem services.
**2**	Examining patterns between socioeconomics and environmental amenities does not capture intent; it is equally important to understand the processes driving inequitable distributions.
**3**	Vegetational structure and social structure may be mismatched. Trees can take a long time to mature while social conditions in a city might change more rapidly.
**4**	Tree canopy cover is treated as homogenous across the unit of analysis however this is unlikely to be the case.
**5**	Trees are not always an environmental amenity.

In this study, we build upon Schwarz et al. (2015) by examining the distributional equity of both urban tree canopy cover (UTCC) and the monetary value of ecosystem services (ESD) provided by urban forests in nine cities from three regions across the United States. We utilized high-resolution socioeconomic and environmental datasets that were obtained from the i-Tree Landscape application and employed both correlation and spatial regression analyses. Using these data and methodologies, we addressed two research questions: 1) Are UTCC and ESD equitably distributed across socioeconomic groups in different urban contexts? and 2) Are the relationships with socioeconomic predictor variables consistent for UTCC and ESD? We predicted that UTCC and ESD would be positively associated with the variables median income and percent with an undergraduate degree and negatively associated with the with the variables percent minority, percent poverty, percent without a high school degree, percent renters, median year home built, and population density (Table 1). We also predicted that there would be differences in the relationships between each response variable (UTCC and ESD) and the suite of socioeconomic predictor variables examined because UTCC solely represents a measurement of canopy extent while ESD incorporates numerous environmental and demographic variables along with UTCC into its calculation.

## Materials and methods

### 1. City descriptions

Nine case study cities from three geographic regions in the United States were selected for our analysis. They included New York City, Philadelphia, and Washington, D.C. from the East Coast, Chicago, Cleveland, and Pittsburgh from the Midwest, and Los Angeles, Sacramento, and San Diego from the West Coast. City selection aimed to identify three large urban areas (with 2010 populations greater than 250,000) within each geographic region for which high-resolution UTCC data were available at the level of U.S. census block group (CBG; unit of analysis) in i-Tree Landscape (see below). Socioeconomic (Table 3) and environmental (Table 4) characteristics varied across the cities and regions.

**Table 3:** Socioeconomic and demographic characteristics of case study cities and regions.

		2010 Population	Population Change from 1950	Median Income	Percent Poverty	Percent Minority	Percent without a High School Degree	Percent with an Undergraduate Degree	Median Year Home Built	Percent Renters	Population Density (#/km)
**East**	New York	8193703	5.3	56396	18.6	55.0	7.3	38.3	1949	66.9	24.7
	Philadelphia	1528271	-26.2	38082	26.0	62.0	3.9	26.2	1947	44.7	8.8
	Washington	605040	-24.6	69622	18.1	62.8	3.2	50.1	1951	53.6	7.3
	Region Average	3442338	-15.2	54700	20.9	59.9	4.8	38.2	1949	55.1	13.6
**Midwest**	Chicago	2697661	-25.5	49565	21.1	56.8	6.7	35.5	1948	53.3	8.2
	Cleveland	395978	-56.7	29373	31.5	65.4	3.3	17.6	1944	54.3	2.9
	Pittsburgh	305405	-54.9	39533	22.3	36.4	1.7	38.6	1945	49.2	3.3
	Region Average	1133015	-45.7	39490	25.0	52.9	3.9	30.6	1946	52.3	4.8
**West**	Los Angeles	3796060	92.7	57366	18.7	49.2	13.4	35.1	1957	58.0	6.4
	Sacramento	467382	239.7	51605	18.2	50.5	7.8	36.4	1967	50.5	2.5
	San Diego	1306176	290.6	66717	14.4	38.2	6.0	46.7	1970	51.2	4.1
	Region Average	1856539	207.7	58563	17.1	46.0	9.1	39.4	1965	53.2	4.3

Values are based on the mean value of census block groups included for each city (with the exception of 2010 P and PC 1950). Data for 2010 P and PC 1950 came from http://worldpopulationreview.com/; data for all other variables came from i-Tree Landscape and are for the year 2010. Region averages are calculated as the average of the three city summary values (versus average of all census block groups across cities).

**Table 4:** Environmental characteristics of case study cities and regions.

		Mean Annual Temperature (°C)	Mean Annual Precipitation (mm)	Mean UTCC (%)	Carbon Sequestration (USD/year)	Total Air Pollution Removal (USD/year)	Avoided Runoff (USD/year)	cbgESD (USD/year)	Mean CBG Size (km)	ESD (USD/year)
**East**	New York	13.0	1268	16.8	418	11321	1559	13297	112	133.6
	Philadelphia	13.0	1055	17.7	1409	15232	3499	20139	234	54.4
	Washington	14.5	1009	26.9	3816	20405	5332	29553	368	65.3
	Region Average	13.5	1111	20.5	1881	15653	3463	20996	238	84.4
**Midwest**	Chicago	10.0	937	19.8	866	10304	3030	14200	282	52.9
	Cleveland	10.5	994	21.4	1644	9439	2955	14037	442	35.0
	Pittsburgh	11.0	970	37.7	3351	19489	6646	29486	399	63.9
	Region Average	10.5	967	26.3	1954	13077	4210	19241	374	50.6
**West**	Los Angeles	18.5	326	11.4	1426	9895	1977	13297	488	24.1
	Sacramento	16.0	470	18.0	1962	8330	1131	11423	857	18.5
	San Diego	17.5	263	12.3	1068	11803	2776	15646	738	17.5
	Region Average	17.3	353	14	1485	10009	1961	13455	694	20.0

**Table 4 Note:** Values are based on the mean value of census block groups included for each city (with the exception of Mean Annual Temperature and Mean Annual Precipitation). Temperature and precipitation data came from https://www.currentresults.com/Weather/US/average-annual-temperatures-large-cities.php; data for all other variables came from i-Tree Landscape and are for the year 2010. Region averages are calculated as the average of the three city summary values (versus average of all census block groups across cities).

On the East Coast, case study cities ranged in population from over 8,000,000 (New York City) to under 1,000,000 (Washington, D.C.) (Table 3). Two of the three cities experienced population decline since the 1950’s while New York City experienced minor growth (Table 3). Cities within the East Coast region had intermediate average values for most of the socioeconomic characteristics examined relative to the other two regions with the exception of the variables percent minority, percent renters, and population density, which were each the highest of the three regions (Table 3). Each of the three East Coast study cities occur within the Eastern Temperate Forest ecoregion and could therefore be expected to have had high levels of canopy cover historically [64]. Furthermore, in the absence of active land management, plant communities in these cities are likely to revert to a forested state on their own. On average, these cities received the most rainfall of any region and had higher average annual temperatures than those in the Midwest yet less average canopy cover per CBG (Table 4).

Many cities in the Midwest of the United States have experienced protracted population decline over the last several decades, and this pattern can be seen with each of the three case study cities in this region (Table 3) [58,65]. Severe urban population decline often results in the proliferation of minimally managed vacant land, which can serve as suitable habit for the establishment of spontaneous vegetation communities that in time can increase UTCC [9,66]. When considering that each of the Midwestern study cities also occur within the Eastern Temperate Forest ecoregion and have historically been heavily forested, this phenomenon might be contributing to these cities having the most UTCC per CBG of any region examined (Table 4) [64]. Numerous cities in the Midwest have also faced substantial economic hardship and as a result, CBGs from the region had the lowest median income, highest percent poverty, and lowest percent with an undergraduate degree on average (Table 3).

The West Coast study cities were the warmest based on mean annual temperature while also receiving the least precipitation (Table 4). Each of the cities in this geographic area occurs within the Mediterranean California ecoregion where warmer temperatures and less rainfall are to be expected relative the Eastern Temperate Forest ecoregion [64]. This dearth of rainfall is likely a significant driver of two of the cities in this region, Los Angeles and San Diego, having the lowest mean UTCC of all study cities (Table 4). While plant community succession can lead to increases in UTCC in this ecoregion, it is less likely than in forested ecoregions. As a result, cities in the West Coast grouping may have markedly different urban forests than what might be found across the other six study cities. From a socioeconomic standpoint, the West Coast study cities had the highest median income, lowest percent minority, lowest percent poverty, highest percent with an undergraduate degree, and lowest population density per CBG (Table 3). CBGs in these cities had approximately double the percentage of people without a high school degree compared with the other two regions (Table 3).

### 2. i-Tree landscape

The majority of data utilized in this study were obtained from the publicly available program i-Tree Landscape v4.0.1 (https://landscape.itreetools.org/). The i-Tree platform includes a suite of software programs and tools developed by the i-Tree Cooperative and based on peer-reviewed research that can be used for quantifying the benefits and values of trees (https://www.itreetools.org/). i-Tree Landscape specifically is a web-based application that allows users to explore the tree canopy, land cover, ecosystem services (including their monetary value in USD), and demographic information for a specific geographic location. Users are able to specify the scale at which data are obtained through the web-interface. For example, data can be provided at the scale of U.S. census place or census block group, among other common boundaries. The program also serves as a repository for high-resolution UTCC data from around the country, and users can chose to view canopy cover and ecosystem service estimates based on high-resolution data or National Land Cover Data (2011 or 2001) for a given location and geographic unit (https://landscape.itreetools.org/hires). All data obtained and analyzed from i-Tree Landscape are publicly available and our usage of them are in compliance with i-Tree’s terms of use.

#### A. Socioeconomic data

All socioeconomic data used in the study were obtained through i-Tree Landscape. i-Tree Landscape derives all U.S. population statistics directly from the U.S. Census Bureau (2010 data) and provides them for various geographic units, including CBGs. The socioeconomic variables provided through i-Tree Landscape are only a subset of all variables measured by the U.S. Census. Predictor variable selection for this study was based on those used in similar empirical urban ecology and environmental justice research (Table 1) and what was available through i-Tree Landscape. The full list of socioeconomic predictor variables includes: median income, percent with an undergraduate degree, percent minority, percent without a high school degree, percent poverty, percent renters, median year home built, and population density. While several researchers have examined individual racial/ethnic group variables (i.e. percent Asian, Black, Hispanic) [15], i-Tree Landscape only presents a single metric termed percent minority, which is calculated as one minus the value for the variable percent White. Compared with other environmental justice studies, we do not use certain socioeconomic variables strictly as controls between cities but rather as potentially relevant predictors of UTCC or ecosystem services across distinct geographic areas [15]. Based on the environmental justice literature and in line with our second hypothesis, we consider the eight variables to cluster into two groups (Table 1): those expected to be positively associated with UTCC (median income, percent with an undergraduate degree) and those expected to be negatively associated with UTCC (percent minority, percent poverty, percent without a high school degree, population density, median year home built, percent renters).

#### B. Canopy data

All UTCC data used in the study were obtained through i-Tree Landscape. Any organization can submit high-resolution UTCC data for a geographic area to i-Tree, which then processes it and makes any calculations derived from it publicly available. While the sources of each geographic area’s data are different, i-Tree requires high-resolution submissions to be 10 m or better resolution and to have over 90% accuracy. More information on submission requirements can be found on the i-Tree Landscape High Resolution Land Cover website (https://landscape.itreetools.org/hires). High-resolution UTCC data were obtained for each CBG within a city. If a CBG within a city did not have high-resolution data available (for example, a large portion of the CBG was outside the city boundary), no data (including socioeconomic) was exported for that CBG and it was excluded from subsequent analyses. Because the variable UTCC is a percentage, we consider it a CBG-size-controlled variable; in other words, the coverage value can be compared across CBGs of varying sizes.

#### C. Ecosystem service calculations

All ecosystem service estimates and their monetary values were obtained through i-Tree Landscape. Trees provide many ecosystem services, including carbon sequestration and storage, atmospheric pollutant removal, and stormwater runoff reduction [7,9]. The i-Tree Landscape program uses tree and impervious surface cover data along with local county data to estimate the annual quantity and monetary value (USD) of the following ecosystem services: carbon sequestration, atmospheric pollutant removal, and stormwater runoff reduction provided per CBG on an annual basis. Because each service has an associated monetary value, the monetary values can be aggregated into a single variable that serves as an indicator of the magnitude of regulating ecosystem services provided. More information on how each service and its monetary value are calculated can be found on the i-Tree Landscape website (https://landscape.itreetools.org/references/data/). Importantly, parameters such as land cover classification, total carbon storage, net annual sequestration, local tree cover, leaf area index, percent evergreen, weather, pollution, transpiration, precipitation interception, avoided runoff, and population data all contribute to the models that generate state- or county-specific estimates that influence ecosystem service calculations. The dollar values provided by each service were then summed to create a single estimate of the monetary value (USD) provided by the forest within a CBG on an annual basis (variable: census block group ecosystem service dollars; cbgESD). Because a larger CBG has the potential to have more forest and thus yield more monetary value than a smaller CBG with the same UTCC level, each summation was divided by the respective CBG area to create a new variable describing the density of ecosystem service dollars provided by a kilometer of land within a CBG on an annual basis (variable: ecosystem service dollars per square kilometer; ESD). This calculation assumes that UTCC is evenly distributed throughout a CBG, which is unlikely to be the case for the majority of CBGs (Table 2). However, controlling for the size of CBGs is necessary in order to make comparisons between them. Our analyses examined ESD, controlling for CBG size, as well as the individual monetary values of carbon sequestration, atmospheric pollutant removal, and stormwater runoff reduction, which did not account for differences in the size of each CBG.

#### D. Limitations

While i-Tree Landscape is a valuable tool for large-scale ecosystem service quantification, it is not without limitations and uncertainties. A detailed discussion of these can be found on the i-Tree Methods and Files website (https://www.itreetools.org/support/resources-overview/i-tree-methods-and-files) under the i-Tree Landscape Resources section. Importantly, the outputs provided by the program are ultimately estimates based on available data and mathematical relationships involving various assumptions. These estimates can be expected to change as data sources and computational methods evolve over time. For example, the majority of i-Tree Landscape ecosystem service estimates for the United States are based on tree canopy cover data obtained from the National Land Cover Database. While geographically comprehensive, the comparatively low resolution of the data (30 m) results in a higher margin of error within spatially heterogeneous urban areas. A growing number of local and regional organizations are acquiring high-resolution spatial datasets for their city or municipality, and when provided to i-Tree, are allowing for more accurate estimates from the program. Users should be aware of the limitations and uncertainties associated with i-Tree Landscape estimates when considering the results discussed herein.

### 3. Geographic data and data processing

Shapefiles containing GIS data layers of census places and census block groups for the nine case study cities were obtained from the U.S. Census TIGER (Topographically Integrated Geographic Encoding and Referencing System)/Line Geodatabases website (https://www.census.gov/geographies/mapping-files/time-series/geo/tiger-line-file.html) for the year 2010. In ArcGIS, a layer of a larger geographic area (typically a county or state) containing all CBGs was clipped to the extent of a census place (i.e. a study city). As CBGs and census places do not always share the same borders, portions of some CBGs were clipped off. The remaining polygons and any associated data were deleted for any CBGs that were over 50% outside of the boundaries of a city. Associated with each CBG is a unique ‘GEOID’ code provided by the U.S. Census. Exported data from i-Tree Landscape (socioeconomic, canopy, ecosystem services) for each CBG were joined to the shapefile data using this unique identifier. If no high-resolution tree canopy data were available for a CBG (see above), the CBG polygon was deleted from that city’s layer. Because ecosystem services are benefits that humans receive from ecological functions, for a CBG to be included, it had to have a population density greater than zero as well as a non-zero value for median year home built. These requirements led to the removal of a small number of the original number of records (less than five percent for New York City and Pittsburgh, less than three percent for San Diego, less than one percent for every other city).

### 4. Statistical analyses

#### A. Partial least squares correlation: Are environmental and socioeconomic variables correlated?

The primary objective of this research was to investigate the associations between socioeconomics, urban forest canopy cover abundance (UTCC), and ecosystem services (ESD) across cities and regions. We began by examining correlations amongst the predictor and response variables. Partial least squares correlation (PLSC) was used to elucidate patterns among the full set of predictor and response variables concurrently. Predictor variables included the eight socioeconomic variables discussed above. Response variables included UTCC, ESD, and the value (USD) of the ecosystem services carbon sequestration, total air pollution removal, and avoided stormwater runoff provided per CBG. PLSC, or canonical partial least squares as it is also known, is a multivariate correlational technique used to analyze associations between two sets of data simultaneously [67–69]. PLSC operates by creating a new set of variables (one latent variable from each set of data) from linear combinations of the original variables. These latent variables are required to explain as much covariance between the two data sets as possible. The procedure was carried out with the function *pls* (mode = canonical) in the ‘mixOmics’ package [70] in R [71]. Next, the *cim* function in ‘mixOmics’ was used to calculate a pair-wise similarity matrix with the similarity value between a pair of variables representing a robust approximation of the Pearson correlation. The function then produces a Clustered Image Map (CIM, also known as a heatmap) depicting the similarity values between all variable pairings. Variable pairs with a similarity value greater than or equal to 0.5 but less than 0.75 were marked with one ‘*’ on the CIM; pairs greater than or equal to 0.75 but less than or equal to 1 were marked with ‘**’. The same thresholds (but negative) and markings were used for negative correlation values.

#### B. Spatial autoregression: When accounting for spatial structure, do socioeconomic variables explain UTCC and ESD?

We also sought to examine the relationship between the socioeconomic variables and each of the focal response variables (UTCC and ESD) when explicitly accounting for the spatial structure of the datasets. Based on previous research, multivariate ordinary least squares (OLS) regression was hypothesized to be an unsuitable statistical method due to the presumed presence of spatial autocorrelation [15,18,37]. Spatial autocorrelation describes the degree to which systematic spatial variation exists for a variable [72]. When spatial autocorrelation is present, additional steps must be taken to avoid biasing the coefficient estimates that would otherwise be obtained from a traditional OLS approach.

In order to first examine the extent to which spatial autocorrelation might be present, we began by creating a queen contiguity-based spatial weight matrix in the order of one for each city using the software program GeoDa v1.12.1.161 [73]. Two OLS models were then developed for each city. Both included seven of the eight socioeconomic variables described above while one used UTCC as the response variable and the other used ESD. Because the calculations that yield the value for ESD incorporate population density, the variable was omitted from all subsequent regression models. Among the outputs provided by GeoDa when running an OLS regression are an Akaike Information Criterion (AIC) value and diagnostics for multicollinearity. The multicollinearity condition number was above the threshold value of 30 for each of the 18 regression models (S1 Table) [74]. This indicates the presence of collinearity among our variables [74], a feature that has been observed in similar studies utilizing several socioeconomic predictor variables simultaneously [15]. Additionally, when running an OLS regression with a weight matrix available, diagnostic indicators of spatial dependence are provided as an output. For each model, Moran’s *I* values were assessed and were found to be significant at a p-value of less than 0.001, indicating strong spatial autocorrelation (S1 Table).

When spatial autocorrelation is present, spatial autoregressive (SAR) models generally represent a superior alternative to OLS regression [15]. Two increasingly utilized spatial autoregressive techniques include spatial error (SEM) and spatial lag (SLAG) models [15,75]. In this study, SEM and SLAG models are used as an alternative to OLS regression in an effort to account for the presence of spatial autocorrelation. The SEM model functions by incorporating spatial effects into the error terms, thereby attempting to account for the spatial structure of unobserved variation. The SLAG model, on the other hand, introduces a spatially lagged dependent variable. Using GeoDa, SEM and SLAG models identical to the aforementioned OLS models were developed examining the effects of the full suite of predictor variables on the two response variables. In all cases, SEM and SLAG models had lower AIC values than their OLS counterparts, indicating an improved fit when accounting for the spatial structure of the data. Additionally, both SAR models include an additional spatial coefficient (Lambda for SEM and Rho for SLAG), which was highly significant in all SEM and SLAG models.

## Results

### 1. Partial least squares correlation: Are environmental and socioeconomic variables correlated?

**Fig 1:**
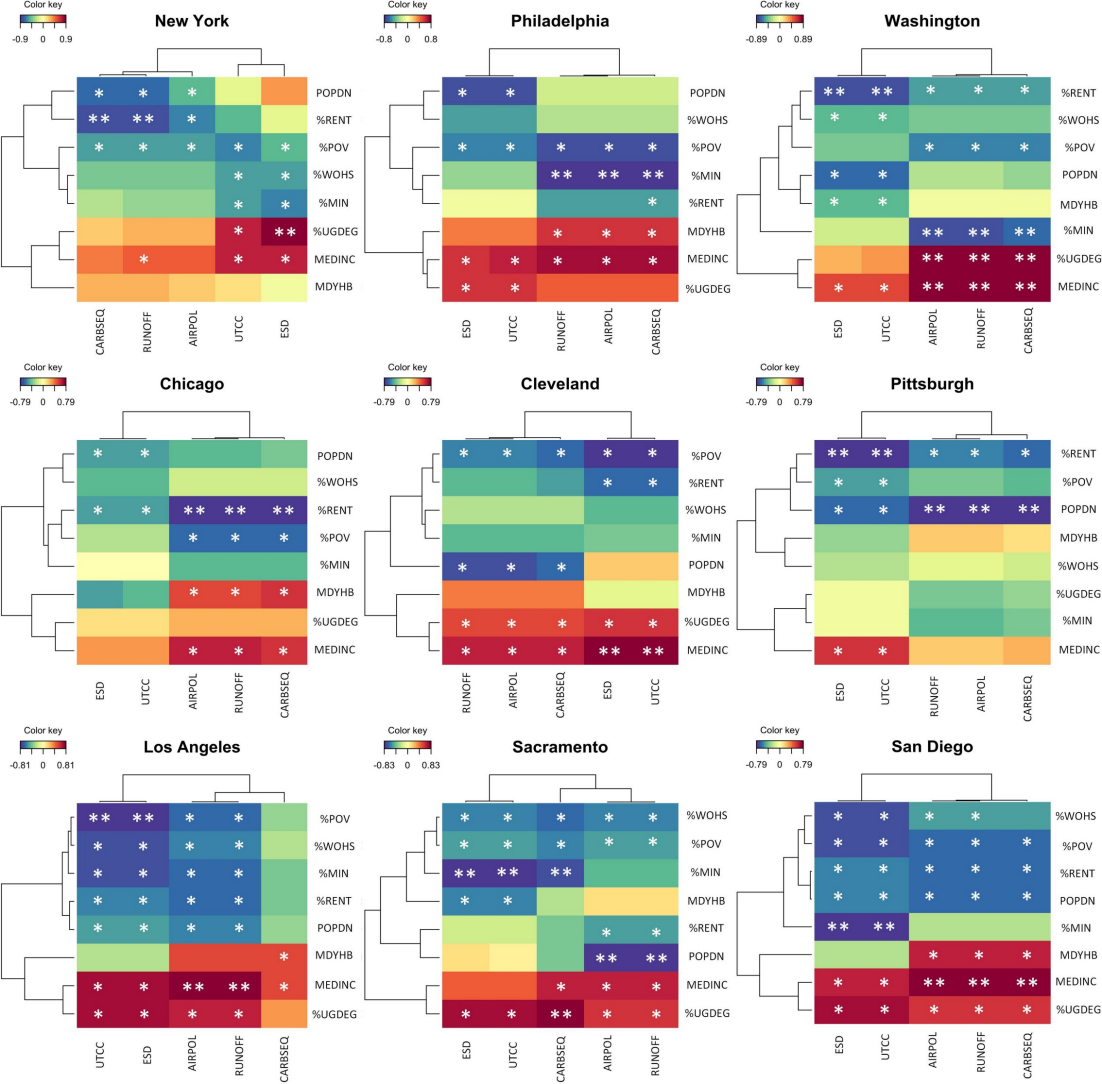
Clustered image maps illustrating partial least squares correlation analysis results for each city. Cells shaded with warm colors (red, orange) have positive correlations while cells shaded with cool colors (blue, green) have negative correlations. Variable pairs with a correlation value greater than or equal to 0.5 but less than 0.75 are marked with one asterisk; pairs greater than or equal to 0.75 but less than or equal to 1 were marked with two. The same thresholds (but negative) and markings are used for negative correlation values.

Results from the PLSC analysis reveal correlations that largely align with our predictions (Fig 1). The socioeconomic variables median income and percent with an undergraduate degree were fairly consistently positively correlated with UTCC and ESD as well as the monetary values of the three ecosystem services examined individually (Fig 1). The variables often used to describe disadvantaged or marginalized populations (percent minority, percent poverty, percent without a high school degree, and percent renters) were generally neutral to negatively correlated with UTCC and its benefits. The correlations between median year home built and population density exhibited some of the greatest variability with the response variables both within and across cities. For instance, median year home built was positively correlated with the full set of environmental variables in New York City and Philadelphia, while it was negatively correlated with UTCC and ESD and positively correlated with the economic values of the carbon sequestration, atmospheric pollutant removal, and stormwater runoff reduction in Chicago and San Diego. Population density displayed the opposite pattern. The variable was often negatively correlated with the full set of environmental variables, but in some cities, population density was positively correlated with UTCC and ESD and negatively correlated with the values derived from the individual ecosystem services. Interestingly, despite the fact that population density is one of the many variables included in the calculation of ESD (but not UTCC), correlation values between the predictor and the two response variables were similar. In most cases, the response variables that controlled for CBG area (UTCC and ESD) and those did not (individual ecosystem service monetary values) had similar correlations with each socioeconomic variable, however some variability did exist and was more pronounced in some cities (e.g. New York City) compared with others (e.g. San Diego).

### 2. Spatial autoregression: When accounting for spatial structure, do socioeconomic variables explain UTCC and ESD?

Across most models, the direction of relationships between predictor and response variables within the SEM and SLAG models were congruent, although the level of significance varied (Table 5). For example, in New York City, percent poverty was positively related to UTCC across SAR models but at p < 0.05 (SEM) versus p < 0.01 (SLAG). In some instances, however, results deviated substantially between spatial models. In Chicago, percent with an undergraduate degree and percent minority were both significant predictors of UTCC and ESD in the SLAG models, but not in the SEM models (Table 5).

**Table 5:** Spatial autoregressive (SAR) model results using urban tree canopy cover (UTCC) and ecosystem service dollars (ESD) as response variables.

				MI	PP	PM	WOHS	WUDG	MYHB	PR	L/R	ΔAIC
**East**	New York	SEM	UTCC	(**)	*	ns	(***)	ns	ns	(***)	***	-1845.5
			ESD	ns	**	ns	(**)	ns	ns	ns	***	-2486.0
		SLAG	UTCC	(***)	**	ns	(***)	ns	ns	(***)	***	**-1910.7**
			ESD	ns	***	ns	(**)	***	ns	ns	***	**-2542.2**
	Philadelphia	SEM	UTCC	**	ns	(*)	ns	**	**	ns	***	-887.5
			ESD	**	ns	(*)	ns	**	**	ns	***	-928.3
		SLAG	UTCC	**	ns	ns	ns	**	***	ns	***	**-897.4**
			ESD	**	ns	ns	ns	**	***	ns	***	**-937.2**
	Washington	SEM	UTCC	ns	ns	ns	ns	ns	ns	ns	***	-300.4
			ESD	ns	ns	ns	ns	ns	ns	ns	***	-326.7
		SLAG	UTCC	ns	ns	ns	ns	ns	ns	ns	***	**-309.8**
			ESD	*	ns	ns	ns	ns	ns	ns	***	**-337.4**
**Midwest**	Chicago	SEM	UTCC	Ns	ns	ns	ns	ns	(***)	(***)	***	-979.0
			ESD	Ns	ns	ns	ns	ns	(***)	(***)	***	-1008.1
		SLAG	UTCC	Ns	ns	*	(*)	*	(***)	(***)	***	**-1016.1**
			ESD	*	ns	*	(*)	*	(***)	(***)	***	**-1046.0**
	Cleveland	SEM	UTCC	Ns	ns	Ns	Ns	***	ns	ns	***	-100.3
			ESD	Ns	ns	*	Ns	***	ns	ns	**	**-103.7**
		SLAG	UTCC	Ns	ns	Ns	Ns	***	ns	(*)	***	-100.4
			ESD	Ns	ns	Ns	Ns	***	ns	Ns	***	-102.5
	Pittsburgh	SEM	UTCC	Ns	ns	*	(*)	Ns	**	(***)	***	-117.8
			ESD	Ns	ns	*	(*)	Ns	*	(***)	***	-126.4
		SLAG	UTCC	Ns	ns	***	(*)	Ns	*	(***)	***	**-121.1**
			ESD	Ns	ns	***	Ns	Ns	*	(***)	***	**-130.8**
**West**	Los Angeles	SEM	UTCC	***	ns	(***)	(*)	*	(*)	(***)	***	**-1121.7**
			ESD	***	ns	(***)	(*)	*	(*)	(***)	***	**-1217.1**
		SLAG	UTCC	***	***	(***)	(*)	ns	(***)	(***)	***	-1091.7
			ESD	***	***	(***)	ns	ns	(***)	(***)	***	-1190.7
	Sacramento	SEM	UTCC	Ns	ns	(**)	ns	**	(***)	*	***	**-167.6**
			ESD	Ns	ns	(***)	ns	**	(***)	ns	***	-114.4
		SLAG	UTCC	Ns	ns	ns	ns	**	(***)	ns	***	-162.7
			ESD	*	ns	ns	ns	**	(***)	ns	***	**-124.3**
	San Diego	SEM	UTCC	**	ns	(***)	ns	ns	ns	ns	***	-190.6
			ESD	*	ns	(***)	ns	ns	ns	ns	***	-196.1
		SLAG	UTCC	***	ns	ns	ns	ns	ns	(**)	***	**-202.4**
			ESD	**	ns	ns	ns	ns	ns	(**)	***	**-206.2**

**SEM**: Spatial Error Model; **SLAG**: Spatial Lag Model; **UTCC**: Urban Tree Canopy Cover; **ESD**: Ecosystem Service Dollars per square kilometer; **MI**: Median Income; **PP**: Percent Poverty; **PM**: Percent Minority; **WOHS**: Percent without a High School Degree; **WUDG**: Percent with an Undergraduate Degree; **MYHB**: Median Year Home Built; **PR**: Percent Renters; **PD**: Population Density; **L/R**: Lambda or Rho, Spatial Coefficients; **Delta AIC**: SAR AIC value minus OLS AIC value. Red cells with no parentheses around asterisks indicate positive relationships; Blue cells with parentheses around asterisks indicate negative relationships. Yellow highlighted and bolded cells in the delta AIC column indicate the SAR model with the greatest decrease in AIC value between equivalent SEM and SLAG models. All SAR models had lower AIC values relative to OLS models.

* denotes p < 0.05

** denotes p < 0.01

*** denotes p < 0.001.

When examining the relationship between the full suite of socioeconomic variables and each response variable, the patterns were far more variable across cities than the results from the correlation analysis indicated (Fig 1, Table 5). With the exception of New York City, the variables median income and percent with an undergraduate degree, when significant, were positively related to both UTCC and ESD. Conversely, with the exception of Sacramento, the variables percent without a high school degree and percent renters, when significant, were negatively related to both response variables (Table 5). The variable percent minority varied noticeably by region; in the Midwest, significant relationships were positive while the pattern was reversed when the variable was a significant predictor for the East and West Coast study cities. Median year home built had significant positive and negative relationships with the response variables in approximately the same number of cities, and the directionality of the patterns was consistent across response variables and SAR models for each city. Percent poverty was not a consistent predictor of either UTCC or ESD, having a positive relationship in just two of the nine cities. The most consistent pattern was the spatial coefficient, denoted Lambda in SEM and Rho in SLAG. This variable was consistently positive and highly significant, indicating the usefulness of the SAR models in accounting for spatial autocorrelation. Additionally, the consistently lower AIC value for the SAR models versus the OLS models again indicates the superior performance of the spatial models. When using the same type of spatial model, results were generally consistent whether examining UTCC or ESD, however, there were a few deviations. For example, median income was negatively related to UTCC but had no relationship with ESD in New York City (Table 5).

## Discussion

Increasing the extent of urban forests while preventing the loss of existing canopy cover are viewed as key sustainability initiatives for municipalities given the multitude of benefits trees provide [54,76–79]. Interdisciplinary research efforts have sought to understand how urban forests can influence the well-being of residents and contribute to urban ecosystem health [4,11,80]. Ecologists, for example, might focus on how forest structure translates to ecosystem functions that in turn yield ecosystem services [9,81], while social scientists might center their attention on the distributional equity of the forest as an environmental amenity [16,18]. With the ongoing development and advancement of ecosystem service modeling tools and technologies, researchers from across disciplines can begin to move beyond the use of tree canopy cover as a proxy for urban forest amenities and begin to examine the distributional equity of ecosystem services. In this study, we employed an integrated approach to answer two guiding research questions: 1) Are UTCC and ESD equitably distributed across socioeconomic groups in different urban contexts? and 2) Are the relationships with socioeconomic predictor variables consistent for UTCC and ESD? We determined that the patterns between UTCC and ESD and the socioeconomic predictors varied markedly depending on the statistical analysis used. When we accounted for the spatial structure of the data and examined all socioeconomic variables simultaneously, we observed substantial variability in their significance as predictors of either UTCC or ESD. This is contrary to what is often suggested within the environmental justice literature, wherein persistent patterns have generally been reported (Table 1). We also noted that although patterns were largely the same between the predictor variables and the response variables UTCC and ESD, deviations did occur. These findings not only have important implications for future research investigating the distributional equity of environmental amenities, but also for natural resource managers and policymakers investing in urban sustainability initiatives.

### 1. Are UTCC or ESD equitably distributed across socioeconomic groups in different urban contexts?

A key goal of this study was to compare the relationships between socioeconomic variables and the response variables UTCC and ESD in different urban contexts. To examine these relationships, we utilized both correlation (PLSC) and regression (SAR) analyses. Importantly, we found inconsistencies in the results of these approaches, which have implications for future distributional equity research. First, the conclusions that could be drawn about the relationships with the socioeconomic predictors varied between the correlation and regression analysis because of inconsistencies in the degree of significance (using a threshold of ± 0.5 for correlations, p-value < 0.05 for regressions), and in some cases, changes in the direction of the response between analyses. Second, when performing regressions, spatial models were consistently more robust than OLS regression models as evidenced by lower AIC values and consistently significant spatial coefficients. Finally, although the results between the SAR models were largely congruent, some variability did occur. These findings highlight the need to move beyond correlation analysis when attempting to understand relationships between variables distributed in space and point to the utility of SAR models as one such tool to do so.

Regarding the socioeconomic predictors, we expected that the variables median income and percent with an undergraduate degree would be positively associated with the variables UTCC and ESD based on the large body of literature that has reported similar associations (Table 1). This prediction was mostly supported by the PLSC results, wherein most cities had strong, positive correlations between these predictors and UTCC and ESD. However, when incorporating the other predictor variables and accounting for the spatial structure of the data through the SAR models, both median income and percent with an undergraduate degree were significant positive predictors of UTCC and ESD in fewer cities. In one instance (New York City), while median income had a strong, positive correlation with UTCC, that relationship was reversed in the SAR models. Interestingly, in four of the nine study cities (Washington, D.C., Cleveland, Pittsburgh, and San Diego), either one or both of these predictor variables had no significant relationship with either UTCC or ESD across SAR models. These findings suggest that despite a large body of research that has frequently reported positive associations between measures of socioeconomic advantage (i.e. wealth and education) and UTCC (Table 1), these relationships are not always consistent across disparate urban settings.

Likewise, our second prediction that variables associated with disadvantaged or marginalized groups and those describing the concentration of residents and neighborhood age would be negatively associated with UTCC and ESD was based on prevailing trends reported in the literature (Table 1). While the variables percent minority, percent poverty, and percent without a high school degree carry inherent connotations of community disadvantage based on historic socioeconomic conditions in the United States, percent renters has a greater degree of ambiguity with regards to what high or low values signal about community status. In some urban contexts, affluent communities can be composed primarily of rental units, while in others it is only low-income neighborhoods where renters abound. However, as Heynen et al. (2006) highlight, renters “also have less direct ability and incentive to invest in the planting and maintenance of trees on rental property”, and it is because of this that we consider them to be disadvantaged in terms of the lack of agency they possess over the forest community occurring on the land they inhabit [16]. Similarly, high or low values for population density and median year home built do not carry inherent connotations of community advantage or disadvantage, and it is possible for a densely populated urban area to be affluent or poor, just as it is possible for older homes to be well maintained and valuable or in various states of disrepair. Correlation analysis revealed that the variables percent minority, percent poverty, percent without a high school degree, percent renters, population density, and median year home built were generally neutral to negatively correlated with UTCC and ESD across cities. When significant in the SAR models, the direction of these patterns was largely consistent for percent renters and percent without a high school degree, mixed for percent minority and median year home built, reversed for percent poverty. Our results again suggest notable inconsistencies in the universality of patterns of inequity across socioeconomic groups occurring in different urban socio-ecological systems. Further, even when patterns of inequity exist for a given variable (e.g. percent renters), it may still be difficult to infer what the implications of that inequity are.

### 2. Are the relationships with socioeconomic predictor variables consistent for UTCC and ESD?

Overall, the relationships observed between UTCC and ESD and the socioeconomic predictor variables were fairly consistent across statistical analyses. The heat maps displaying the results from the correlation analysis reveal a high level of similarity in correlation values between UTCC and ESD for a given socioeconomic variable, as indicated by the color of the cell. Similarly, the majority of the relationships and even their degree of the significance are the same between models utilizing UTCC or ESD as the response variable in the SAR models. Given i-Tree Landscape’s emphasis on regulating ecosystem services, the high degree of similarity between the two response variables is expected. For these services, increases in UTCC should correspond with increases in the size of the biogeochemical pools and fluxes associated with it. Although we examined just a subset of the regulating ecosystem services provided by urban forests, we predict that many additional benefits such as temperature regulation and noise reduction (and their monetary value) would scale with increases in UTCC in a predictable and similar manner.

While UTCC and ESD appeared to be highly similar variables, they were different enough to result in some variation in their relationships with the socioeconomic predictors examined within PLSC and SAR models. For example, in New York City, there were several instances where the correlation values differed between UTCC and ESD for a given socioeconomic predictor variable. Similarly, the SAR model results for Cleveland showed that the variable percent minority was a significant predictor of ESD but not UTCC (SEM) and that the variable percent renters was a significant predictor of UTCC but not ESD (SLAG). These findings suggest that while UTCC may be an excellent proxy for many of the benefits provided by trees, it is ultimately not equivalent, and important variation may exist. What’s more, the utility of the variable may be diminished further when considering additional ecosystem services not included in this study. For instance, services such as the recreational value of the habitat or its value for wildlife are likely to be more difficult to quantify. These variables do not necessarily scale with increasing forest coverage, and instead depend on factors such as resident perceptions and cultural values, as well as forest configuration and community composition [25,82–86].Thus, researchers seeking to examine the distributional equity of an environmental amenity such as urban forests should attempt to quantify specific benefits of interest directly when possible, and acknowledge the limitations associated with the variable UTCC cover as a proxy. This is an especially important point when considering issues of environmental justice, as inequities produced by varying levels of UTCC may be more or less pronounced based on the value of the benefits provided by the forest in that geographic area (or, conversely, the costs of the disamenities).

### 3. Additional considerations

Beyond the caveats and limitations mentioned in Table 2, additional considerations are relevant to the study of urban forest distributional equity. First, the absence of a relationship between any given socioeconomic variable and either UTCC or ESD does not guarantee the equitable distribution of that environmental amenity across individuals. As an example, consider the unique context of a shrinking city. In cities that have experienced protracted economic decline, vacant land proliferates within neglected inner-city neighborhoods that are often predominately composed of vulnerable populations [58,87]. In the absence of regular vegetation management and upkeep, the plant communities on vacant land can undergo succession resulting in an increase in UTCC levels [66], particularly in ecoregions where forests typify the climax community. For example, in the shrinking city of Toledo, Ohio, a weakening relationship was observed between housing vacancy and greenness (as measured through the normalized difference vegetation index; NDVI) between 1980 and 2014 [88]. Schwarz et al. (2018) proposed that the change in this relationship over time could be attributable to an increase in spontaneous vegetation in high vacancy areas due to the loss of infrastructure and reduced vegetation maintenance. Such a phenomenon could potentially explain the positive relationships we observed between the variables percent minority and UTCC and ESD in many of the Midwest study cities, patterns that did not exist for study cities on the East or West Coast where shrinkage is not nearly as pronounced if occurring at all. While these findings would suggest that minority populations in these cities might have greater access to UTCC, the reality may be that the green spaces that are dominated by spontaneous vegetation are of low quality or even yield an excess of environmental disamenities [66].

We must also consider the importance of historical social and economic patterns and processes on the distributional equity of tree canopy cover that we see today [89]. Our analysis identified the patterns that could be observed during the snapshot of time when the socioeconomic and environmental data we analyzed were obtained, however it offers no insights into what might have generated them. Unlike herbaceous plant communities, trees and the forest communities they form can take years if not decades to develop, and so the canopy cover levels present today are in many ways a reflection of the planting programs, preferences, and habits of past residents and neighborhoods [21]. The process of “redlining” represents one example of a historic practice applied in neighborhoods across U.S. cities that may have contributed to the distribution of UTCC and ESD that can be observed today [89]. As a form of spatial segregation, the redlining of neighborhoods resulted in long-term patterns of disinvestment and property value stagnation in predominately African American neighborhoods [89]. However, the act of a neighborhood experiencing redlining in the past does not necessarily result in a depauperate forest community at present. The Bolton Hill neighborhood in Baltimore, Maryland experienced redlining beginning in the 1930s, but was selected to undergo redevelopment in the 1960s via a number of urban renewal projects that have resulted in the neighborhood currently having one of the most impressive street tree presences in the city [90]. Thus, the urban forests seen today in cities across the United States are very much a product of the legacies of a variety of social and economic processes of the past.

As a result of these considerations, policy makers and natural resource managers must be aware that even when there appears to be equitably distributed quantities of an environmental amenity such as UTCC, the metric do not capture the full suite of benefits (or conversely, disamenities) that people derive from urban green spaces. When urban planners are aware of the limitations of distributional equity research, it can serve as a useful form of exploratory analysis capable of revealing where inequities might exist across large geographic areas. However, the results should serve primarily as a starting point for local agencies or organizations to further explore the distribution and condition of the natural resources they manage. In doing so, they can seek to better understand how inequities might be amplified or lessened by differences in the quality of the resource of interest (e.g. urban tree canopy cover). Furthermore, when possible, practitioners should also attempt to move beyond the use of proxies for environmental amenities and consider the distribution of the specific ecosystem services (and disservices) that they provide.

## Conclusion

This study utilized an integrated approach to inform urban forest sustainability initiatives. The majority of studies within the field of environmental justice have examined the distributional equity of urban forests using UTCC as a proxy for ecosystem services. With the advent of new methodologies, it is possible to study a subset of ecosystem services directly and quantify their value and distributional equity, which represents an important advancement in the field. When focusing on regulating ecosystem services such as carbon sequestration or air pollution removal that are likely to scale with increasing forest biomass in a predictable manner, measurements of canopy cover extent (i.e. UTCC) may be closely linked with estimates of services or their monetary values (i.e. ESD). However, as additional services are evaluated and quantified, particularly those that might share a more complicated relationship with canopy extent, we expect further deviations between the two variables to occur.

Not surprisingly, we also found that experimental design and the approach taken with statistical analyses can significantly influence the findings and conclusions of distributional equity research studies. Traditionally, researchers in this field have focused on single case studies and utilized correlation analysis [15]. This approach does not allow for the universality of patterns to be examined across disparate urban contexts and fails to consider that socioeconomic data are often spatially distributed, and as a result, spatially autocorrelated. We determined that the conclusions drawn from the correlation analysis differed significantly from those gathered from the SAR models. Further, variation even existed in what socioeconomic variables were significant predictors between the two types of SAR models. Despite this variability, the spatial models represented an improvement over OLS regression in all cases. This finding has been reported by other researchers [15,21,33] and should be considered in both large-scale and case-study investigations of the distributional equity of environmental amenities in the future.

Finally, from our examination of the relationship between eight common socioeconomic predictors, we highlight that substantial variability exists in the patterns of UTCC and ESD distribution across socioeconomic groups. These findings run in contrast to what has frequently been reported in the environmental justice literature. While not universally consistent, most distributional equity studies report positive associations between advantaged communities and access to environmental amenities. Our results therefore serve as a reminder that while environmental inequities often exist, understanding and remedying them will often be dependent on the local context.

## Supporting information

S1 Table OLS model results. The model results of the OLS regressions using UTCC and ESD as response variables.
(XLSX)
