# Correction: Examining the distributional equity of urban tree canopy cover and ecosystem services across United States cities

**DOI:** 10.1371/journal.pone.0230398

**Published:** 2020-03-06

**Authors:** The PLOS ONE Staff

## Notice of republication

This article was republished on February 19, 2020, to correct errors in Fig 1. The publisher apologizes for this error. Please download this article again to view the correct version. The originally published, uncorrected article and the republished, corrected articles are provided here for reference.

## Supporting information

S1 File Originally published, uncorrected article. (PDF)

S2 File Republished, corrected article. (PDF)
